# Potential and challenges of Positron Emission Tomography beyond conventional preclinical and clinical imaging^☆^

**DOI:** 10.1016/j.zemedi.2026.06.001

**Published:** 2026-06-29

**Authors:** Nicola D’Ascenzo, Magdalena Rafecas

**Affiliations:** a Department of Information Science and Technology, University of Science and Technology of China, 96 Jinzhai Lu, Hefei, 230026, Anhui, China; b School of Life Science and Technology, Huazhong University of Science and Technology, 1037 Luoyu Lu, Wuhan, 430074, Hubei, China; c Institut für Medizintechnik, Universität zu Lübeck, Ratzeburger Allee 160, Lübeck, 23562, Germany

**Keywords:** Zebrafish PET imaging, Crop PET imaging, Digital silicon photomultiplier, PET detectors, Mobile PET systems

## Abstract

Positron Emission Tomography (PET) is a non-invasive, highly sensitive functional imaging modality, firmly established in clinical practice for the diagnosis and assessment of therapeutic efficacy in oncological and neurological disorders. Recent advances in digital image processing, sensor technology, and imaging algorithms have enabled this technique to achieve sub-millimetric spatial resolution and a high degree of compactness, thereby making its application to the investigation of biological systems smaller than humans both feasible and scientifically meaningful. In this review, we detail the substantial impact of PET on emerging applications in biomedicine, food security, and agriculture. We focus specifically on non-conventional implementations of PET, including small-animal imaging in species beyond traditional murine models, and imaging of crops. The discussed translational pathways of PET – *from bench to bedside* and *from-lab-to-field* – impose stringent technological requirements on system design and performance, thereby driving further innovation in PET detector technologies and associated electronics.

## 1 Introduction

Positron Emission Tomography (PET) is a non-invasive and highly sensitive technique for measuring biochemical processes in cells and living organisms [1]. PET has been adopted as a major imaging tool for both diagnosis and therapy monitoring across a wide spectrum of human diseases, ranging from cancer to neurological disorders [2], [3], [4], [5].

The PET methodology relies on radiotracers, biochemical compounds that bind to specific cellular processes. These tracers are labeled with positron-emitting nuclides, enabling the reconstruction of a 3D image of their distribution using tomographic techniques. This is achieved by detecting the two collinear 511 keV annihilation rays emitted in the vicinity of the positron decay site [6]. Although tracer development has historically been driven by clinical needs, tracers are intrinsically versatile and can be designed to target functional mechanisms in any living system, beyond humans [7], [8], [9].

Consequently, PET has the potential to become a powerful imaging technique in fields beyond clinical applications, providing quantitative measurements of biochemical and biophysical processes at both cellular and systemic levels—information that is often crucial yet still lacking. In fact, PET already plays an important role in linking basic biomedical research with clinical imaging through dedicated preclinical PET scanners, as the technology is readily scalable from most animal models of human diseases to the clinics, effectively bridging the gap *from-bench-to-bedside* [10]. While rodent models remain central to preclinical research, their high costs, ethical constraints, and reproducibility challenges [11], [12], [13] motivate growing interest in alternative vertebrate models such as zebrafish and chicken embryos. This interest has also translated in analyzing possible issues in reproducibility and developing standardized guidelines, e.g., [14], [15], [16], [17], [18], [19], [20]. In any case, the use of PET for these animals require systems capable of capturing precise biochemical dynamics at the sub-millimetric spatial scales of these tissues of these small animals [21], [22].

Although several studies using chicken embryos have been conducted on conventional preclinical scanners, the spatial resolution of current small animal PET systems limits the applications that involve small anatomical structures [23], [24], [25]. Partial Volume Effects (PVE) become particularly pronounced for objects whose size is less than approximately three times the system’s spatial resolution, measured as the Full Width at Half Maximum (FWHM); given the small dimensions of the embryo organs (few millimeters), such effects are less restrictive in larger animal models such as mice. Hence, further improvements in spatial resolution could significantly enhance the visualization of sub-organ metabolic heterogeneity, tumor perfusion, and potentially cardiac developmental abnormalities.

As PET scanners have become more accessible, PET imaging is attracting interest beyond traditional preclinical and clinical contexts, including veterinary medicine, comparative physiology, ecology, biology and even evolutionary research [26], [27], [28], [29], [30]. Each of these fields can benefit from PET’s ability to non-invasively track biochemical processes in living organisms. Additionally, PET technology is also well suited for in-vitro specimen imaging, providing high-sensitivity, quantitative measurements of tracer distribution in excised tissues and small biological preparations [31].

Beyond biomedical research, PET is also gaining importance in agronomy. Plant cells host a complex network of biochemical processes in response to stress and fertilizers, offering quantitative insights that complement the qualitative data provided by conventional remote sensing methods [32]. Scalable and mobile PET systems, suitable for both controlled laboratory experiments and open-field conditions, bridge the gap *from-lab-to-field*. This enables more sustainable crop management practices, reducing nitrogen pollution while improving yield [33].

Adapting PET to these frontier applications requires systems with sub-millimetric resolution, mobility, low power consumption, and real-time operation. These demands pose challenges not only in sensor design but also in image reconstruction and data processing [34].

In this review, we outline the challenges of the translational pathways of PET in these unconventional fields of application. Section 2 discusses the scientific significance of applying PET beyond its well-established clinical use. Section 3 examines the technological requirements posed by these new applications. Section 4 presents current PET systems and prototypes. Finally, Section 5 provides an outlook on emerging technologies and open scientific questions in this rapidly evolving field.

## 2 Open challenges for functional imaging

### 2.1 Preclinical biomedical research: beyond mice and rats

Animal models are essential tools for studying target expression, imaging agent specificity, metabolism, and pharmacokinetics at both organ and systemic levels [35]. Mammalian models have long been the standard for modeling human diseases, owing to the strong homology between mammalian genomes, anatomy, and physiology [36]. Among these, rodents are most commonly used because they are suitable to genetic modification, relatively easy to maintain, and straightforward to handle [37]. However, preclinical studies in mammalian models often require several months to complete, and animal care entails substantial maintenance costs. Moreover, such experiments must comply with stringent ethical and licensing regulations, further prolonging study timelines—an obstacle for developing rapid therapeutic responses. Consequently, the principles of replacement, reduction, and refinement (3R) underscore the need for alternative, non-mammalian yet biologically relevant animal models [38].

In this context, the zebrafish (*Danio rerio*) has recently gained prominence as a genetically tractable vertebrate model system [39], [40]. The combination of Clustered Regularly Interspaced Short Palindromic Repeats (CRISPR)-based knockout technology and large-scale datasets from next-generation sequencing enables efficient functional validation of Genome-wide association study (GWAS) candidates in zebrafish. This approach makes zebrafish a significant model for clinical research, as it allows the identification of causative genes and molecular mechanisms underlying the pathogenesis of human genetic diseases, which can be investigated using various imaging modalities. The optical transparency of zebrafish embryos and larvae makes optical microscopy a natural choice, including functional imaging approaches. In contrast, adult wildtype zebrafish are optically opaque due to pigmentation, scattering, and increased body thickness, which limits penetration depth and whole-body visualization. In such cases, non-optical modalities such as PET may offer advantages for deep-tissue, whole-body, and quantitative functional imaging. However, bringing PET technology to zebrafish and other small aquatic animals demands substantial efforts and optimization of devices and protocoles. With the aim to determine if fish models are an alternative to mammalian models, a feasibility study of fish glucose metabolism has been carried out using [^18^F]-Fluorodeoxyglucose ([^18^F]-FDG) PET/CT [41]. As this study relied on a clinical PET/CT scanner with limited spatial resolution, the species included were considerably larger than zebrafish. The authors concluded that the considered fish species displayed robust, quantifiable glucose uptake, and their Standardized Uptake Value (SUV) patterns aligned more with humans than with rodents or dogs. In the same line, goldfish were imaged in [42], [43]. Few studies have explored the feasibility of zebrafish PET using commercial small-animal PET scanners, as this application faces specific challenges. Firstly, microinjectors are required if the tracer is to be administered through the caudal vein. An alternative is intraperitoneal (IP) injection [44], [45]. These works were limited to analyze glucose metabolism in healthy adult wildtype zebrafish by means of [^18^F]-FDG PET/CT, either to support models of hyperglycemia in zebrafish [44] or to investigate the feasibility of PET in a brain-dead specimen [45]. A potential risk of this technique is the possibility of tracer accumulation at the injection site, while uptake in other regions remains low or invisible [45]. Dedicated protocols, in which the animals are placed in water after injection and sedated again for scanning, delivered better results [46], [47].

Other tracers tested on zebrafish have been ^18^F-fluoro-6-thia-heptadecanoic acid to study the lipid metabolism in tumors [48], as well as sodium fluoride (^18^F—NaF). The latter should accumulate in bones although uptake in the gills and near the spine was reported [49]. These outcomes underscore the need for further and systematic studies to gain a better understanding of tracer kinetics and animal physiology.

The small size of zebrafish not only requires high-resolution devices, but also high sensitivity, as the amount of radiotracer that can be safely injected into a fish is very limited (IP ≈ 10–20μl /g [50], and 1–3μl /g for injection in the caudal vein [51]).

**Fig. 1:**
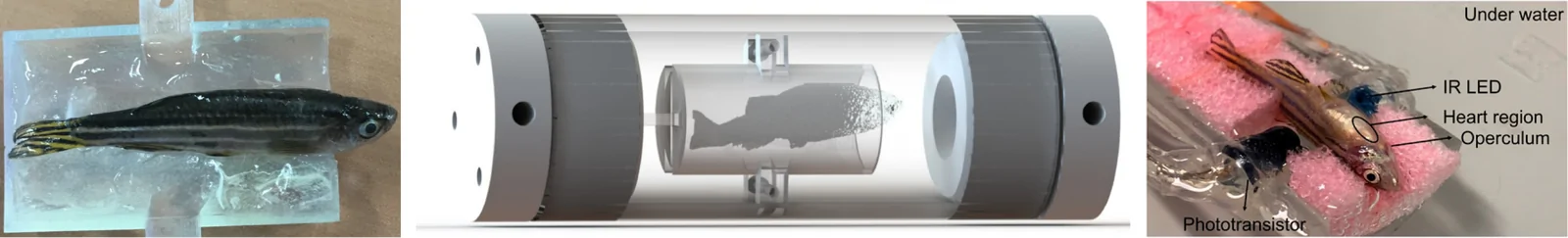
Two possible solutions for fish fixation. Left: Fish in agarose before placement in the water-filled container shown in the central picture. Reprinted from [56], ©2019 IEEE, with permission. Right: Fixation in a sponge, and system for vital signs monitoring. Reproduced from [54], CC BY 4.0.

Depending on the scan time and the specific regulations for animal experimentation, another challenge is maintaining the animals anesthetized, immobilized and within an aquatic environment during imaging, as well as monitoring their vital signs. To address some of all these issues, a variety of solutions have been proposed of different complexity, most of them rely on some kind of water-filled container or imaging chamber [42], [45], [52], [53], [54]. Additionally, further concepts that have been developed for other modalities could be easily transferred to PET, e.g. [55]. Some examples are shown in Fig. 1.

The presence of water in the Field of View (FOV) contributes to image degradation due to attenuation and Compton scattering of the annihilation radiation. As shown in an in-silico study of zebrafish PET [21], this effect is non-negligible but it can be efficiently corrected even with a simple attenuation map of the imaging chamber filled with water. The latter is a feasible solution for standalone PET concepts. While the addition of a Computer Tomography (CT) component can provide more accurate data for attenuation and scatter correction, the information provided by the CT image might be insufficient as anatomical reference. The reason is that even at relative low tube voltages, the organs exhibit minimal density differences; therefore, the images are characterized by low contrast, mainly showing the bone structure and the swimming bladders. However, the use of additional aluminum filters might be sufficient to identify regions of the digestive tract and the heart [57].

Similarly, avian models have gained attention as a viable alternative to mouse xenografts in preclinical cancer research. The fertilized chicken egg provides a unique experimental platform through the chorioallantoic membrane (CAM), a highly vascularized extraembryonic structure that supports rapid and robust tumor engraftment [58]. Unlike rodent models, where tumor establishment and monitoring can take several weeks, the CAM model enables tumor growth within just a few days, offering a markedly faster and more cost-effective approach. Moreover, chick embryos up to embryonic day 14 (E14) are not capable of experiencing pain, allowing their use without the need for formal animal protocol approval [23]. This ethical and practical advantage, combined with the accessibility and transparency of the CAM, has made the model increasingly attractive for oncology studies. Notably, in-ovo PET imaging has emerged as a powerful tool in this context, with successful demonstrations of xenografts implanted on the CAM highlighting its potential for noninvasive cancer imaging and therapeutic evaluation [59], [60]. While the CAM model is inherently limited by a narrow developmental window for investigation (typically 4–5 days for tumor studies to avoid the need for analgesics), its high vascularization and rapid engraftment offer a unique ‘accelerated’ platform for initial therapeutic screening before moving to more complex models.

In addition, invertebrates models are also emerging for potential applications. For instance, snails have been employed to study learning and memory [61], also with support of PET imaging [62]. Also, the cephalopod mollusk *Octopus vulgaris*, proposed as a comparative model for regeneration with relevance to vertebrate systems, has been explored with PET [63].

Ex-vivo PET complements in-vivo imaging of model animals by providing quantitative molecular imaging of isolated organs or tissue samples. Within this field, 3D imaging of micro-scale specimens might be particularly challenging. For instance, the so-called *PET-on-a-chip* approach [64] proposes a miniaturized PET device for imaging organs-on-chips and organoids, thus requiring sub-millimeter spatial resolution and extremely high sensitivity. The final goal is to provide volumetric images. However, if 3D information is not required, 2D autoradiography and radionuclide imaging (beta-imaging) are well-established alternatives which can provide the required resolution. In particular, dedicated devices for positron emission microscopy have been developed for planar (2D) radioisotope imaging of patient-derived organoids [65].

**Fig. 2:**
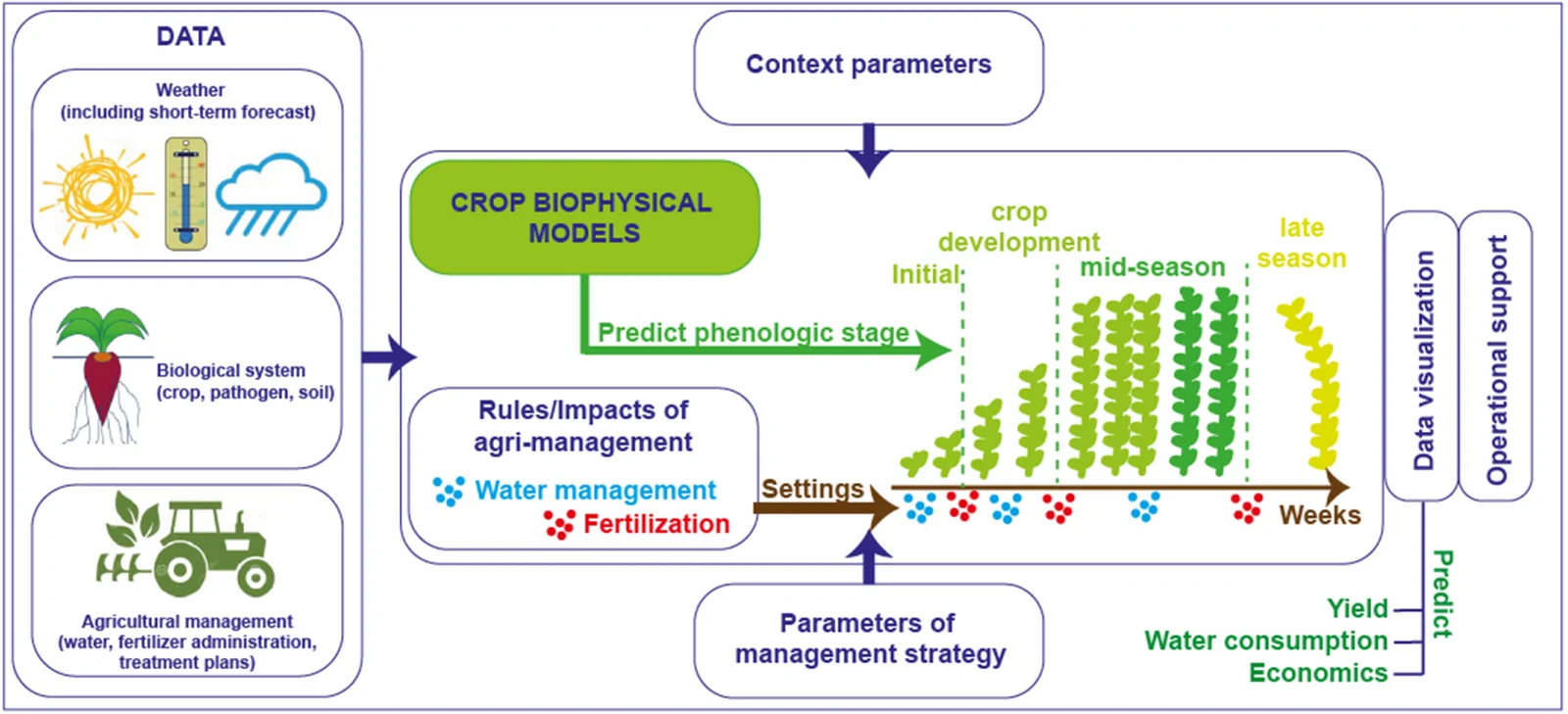
The principle of Decision Support Systems (DSS) and the role of biophysical crop models.

### 2.2 Crop science: beyond laboratory plants

Crop growth models are the essential tools at the basis of Decision Support Systems (DSS), allowing to predict yield and water consumption under specific crop treatment plans, therefore positively impacting into proper N-fertilizers administration and water management [66]. Since their introduction, crop biophysical models, mainly based on weather, soil, and large scale remote satellite imaging data, have become indispensable tools for optimizing agricultural practices and assessing the impact of nitrogen-based fertilizers on predicted yields [67]. The continued advancement of these physically based models has supported farmers worldwide in making informed management decisions—such as determining optimal sowing times, plant population densities, and irrigation regimes—particularly under extreme weather conditions, where water availability and temperature stress remain key limiting factors in crop productivity [68].

The DSS operational principle is represented in Fig. 2. Data collected in field during the agronomic cycle are essential to the understanding and steering of management practice. They include weather forecast, chemical soil composition, humidity, observations of the biological crop system, such as chlorophyll content, color, water content obtained with hyperspectral imaging and remote sensing, and finally treatment, irrigation and N-fertilizer plans. These data represent the main input to crop biophysical models, which predict the phenologic states of crops. In fact N-fertilizer cycles, biostimulants, water management, depend on the proper identification of the phenologic stages [69], [70], [71], [72]. Once merged with context paramenters and with the management strategy parameters, the crop models provide a visualization of the data and a prediction of the yield, water consumption and economical losses related to a treatment plan. The agronomist can therefore arrange the operations in field in correspondence with the expected gain. It is clear that the stability of the crop growth model is essential in order to provide reliable steering quantities. Still many open challenges remain, in particular towards predicting the phenological state with an uncertainty below 6–7 days, limiting the determination of the correct timing for N-fertilizer administration, predicting the physiological impact of N-fertilizers to the plant evolution right after the administration, therefore overestimating the correct quantity of fertilizer to be applied, and quantitative data reflecting the actual physiological and functional status of the crop.

Radiotracers have been employed in plant science since the inception of the field. Investigations of lead translocation in crops were already conducted in the 1930s using thorium, and these studies demonstrated that a radioactive isotope of a given element exhibits essentially the same chemical and biological properties as the corresponding stable nuclide. This finding laid the foundation for the broader application of radiolabeled compounds in both clinical and experimental settings [73], [74], [75]. With the establishment of PET, a variety of radiotracers has been employed for in vivo imaging and quantitative assessment of functional processes in plants. ^11^C—CO${}_{2}$ is typically administered to plants in the gaseous phase, taken up by the leaves, and subsequently metabolized via photosynthesis, leading to the synthesis of glucose, whose transport and assimilation can then be quantitatively investigated. Water labeled with dissolved ^18^F, absorbed directly through the roots, can be used to visualize transport mechanisms and is often preferred over ^15^O—H${}_{2}$O owing to the longer physical half-life of ^18^F compared with ^15^O. These methodologies are usually implemented in laboratory-based experiments with conventional micro PET systems under controlled illumination, temperature, and humidity, which must be maintained stable throughout the measurements because of the intrinsic capacity of plants to adapt dynamically to environmental fluctuations.

Progress towards modeling of functional mechanisms in crops right after treatment has been brought by the development of movable PET, Magnetic Resonance Imaging (MRI), and Optical Endoscopic Microscopy (OM) for in-field use, which offer in-depth insight into the importance of quantitative biophysical transport and metabolic phenomena in crop modeling [76], [77]. The implementation of PET in open-field conditions requires rigorous adherence to regulatory frameworks governing the handling, distribution, and environmental release of radiotracers. To the authors’ knowledge, the first—and thus far only—experimental configuration enabling such studies consists of cultivating crops in dedicated cylindrical vessels, thereby ensuring continuous accessibility of the root system for tracer uptake while maintaining physical isolation of the soil to prevent in situ contamination [76]. The use of low activity levels (on the order of a few tens of microcuries or few hundreds kilobequerel) in combination with the short physical half-lives of the employed tracers substantially limits radiation exposure and ensures safe experimental operation.

The rationale behind the use of imaging in digital precision agriculture is that a series of local and systemic pathways profoundly influence gene expression, metabolism, physiology, growth, and development. Their measurement can predict with precision the phenological state of the crop. Furthermore, N acts as a trigger for these processes. Genetic responses can occur within 5 to 120 min after N application, followed by phytohormone signaling that regulates N availability, plant growth, carbon metabolism, nutrient transport from source to sink, and overall crop development [78]. Despite their significance, current crop models largely neglect these rapid biophysical and metabolic processes due to the absence of suitable input data collected in field together with the traditional agroclimatic, remote sensing and satellite observations. However, integrating such data—when made accessible—would enable early detection of fertilizer treatment effectiveness and support the precise application of nitrogen, thereby reducing waste and enhancing sustainability.

## 3 Technological challenges for new fields of applications of PET

### 3.1 Significance of resolution and sensitivity in novel applications

As depicted in the previous section, the novel applications of PET demand a spatial resolution that falls well below the millimeter scale characterizing existing scanning technologies. Moreover, these applications require a sensitivity level adequate to monitor localized activity at a scale of a few hundreds of kilobequerels per cubic millimeter. The minimal requirements to perform experiments in these novel applications require therefore a setting of spatial resolution and sensitivity which is much below that of PET preclinical or clinical scanners. Clinical PET systems require a minimal spatial resolution of approximately 1.5 mm and must be sensitive to a local activity of approximately $8\times 10^{-3}$ MBq/g. Imaging in mice can be conducted with a higher radiotracer dose, up to approximately $4\times 10^{-2}$ MBq/g, while requiring a lower spatial resolution of approximately 0.9 mm to visualize smaller tissues. Emerging biomedical models utilizing zebrafish and chicken embryos necessitate an enhanced spatial resolution of 0.7–0.8 mm, maintaining a similar average activity concentration with respect to clinical imaging. Finally, plant imaging in field must achieve a spatial resolution of 0.7–0.8 mm with a significantly low activity concentration of $8\times 10^{-3}$ MBq/g, due to regulatory constraints and the low uptake by the thin and soft plant tissues.

It is therefore evident that innovative applications of PET outside conventional fields are driving progress in PET technology to explore possible achievable boundaries, where spatial resolution and sensitivity are inherently interconnected.

The spatial resolution, denoted as (Γ), of a PET system is expressed as follows [79]:

(1) $$\Gamma \;\left[\mathrm{mm}\right]=\alpha \times \sqrt{s^{2}+{\left(\frac{d}{2}\right)}^{2}+\frac{{\left(12.5\;\left[\mathrm{mm}\right]\;r\right)}^{2}}{r^{2}+R^{2}}+{\left(0.0044\;R\right)}^{2}}$$

In this context, the variable s denotes the penetration range of the emitted positron within the biological substrate. The parameter d represents the cross-sectional measurement of the crystalline elements that constitute the PET sensors. The symbol R corresponds to the radius of the PET scanner apparatus, while r signifies the radial position of the object being imaged, located within the designated FOV. Finally α denotes the natural smearing occurring as a result of the image reconstruction algorithm. The unit of length of the variables is in millimeters.

The penetration depth of positrons within soft tissues depends on the kinetic energy of the emitted positrons, which is inherently linked to the specific radionuclide utilized. The mean penetration range in water spans from 0.54 mm for ^18^F to 2.8 mm for ^68^Ga. This causes an irreducible smearing of the measured tracer distribution following a distribution with a relatively sharp peak and long tails. In addition, thin plant tissues exhibit dimensions on the order of a few hundred micrometers, considerably smaller than the positron range. In this case positron range induces a loss in sensitivity due to the escape of positrons from the tissues. Nevertheless, advances in high-resolution PET technology have increasingly motivated research into positron-range correction at the image reconstruction level, or through image processing (e.g., deconvolution, AI-based approaches, etc.) [80], [81], [82], [83].

The spatial resolution in PET systems is predominantly constrained by the size of the scintillation crystals employed. In the context of PET systems specifically engineered for the imaging of small biological specimens such as zebrafish or agricultural samples, it is imperative that the crystal cross-sectional dimension does not exceed 1.5 mm. From the perspective of sensor technology, there currently exists no significant constraint on the dimensions of individual PET sensor units within a pixelated array configuration. Advances in crystal technology have enabled the fabrication of crystal sizes as small as 0.5 mm [84], [85]. Furthermore, the readout of individual crystals can be efficiently accomplished using customized Silicon Photomultipliers (SiPMs) which are produced in cost-effective Complementary Metal–Oxide–Semiconductor (CMOS) foundries. This allows for the creation of sensors of any size compatible with the scale of the node [86], [87]. Nevertheless, this constraint incurs a reduction in sensitivity, attributable to the inherently increased non-sensitive interstitial spaces between individual crystal units when they are arranged in arrays. This phenomenon arises as a consequence of the packaging requirements during the array assembly process. At present, a crystal cross-section measuring approximately 1.5 mm appears to represent the optimal balance between spatial resolution and sensitivity in pixelated sensors utilized in high-resolution dedicated PET scanners.

In addition to pixelated detectors, monolithic crystals present an alternative approach for achieving high spatial resolution. These systems comprise large crystals, with dimensions reaching up to 5 cm, which are interfaced with an array of sensors. The detected light patterns at these sensors are associated with the γ ray impact points within the crystal, and these points can be reconstructed with millimetric precision [88], [89]. Algorithms based on Convolutional Neural Networks (CNNs) have been developed to facilitate the reconstruction of the impact points from the light distribution captured by the sensor arrays. Nevertheless, count rate performance remains a challenge in these detection units due to the substantial size of the scintillator, which results in a higher count rate and increased susceptibility to pile-up effects. This issue is of particular importance for non-pure positron emitters (${}^{124}I$, ${}^{89}\mathrm{Zr}$, ${}^{64}\mathrm{Cu}$, etc.) that emit additional prompt or cascade γ rays, which increase the probability of pile-up in monolithic detectors.

The influence of the Depth of Interaction (DOI) on spatial resolution is encapsulated within the third term of Eq. (1). Imaging systems specifically engineered for small biological specimens, such as plant models, organoids, or zebrafish, generally exhibit a reduced radius R and an elongated shape. This combination exacerbates the effects of the parallax error caused by the missing information about the DOI, thereby degrading image quality, particularly in the radial direction, as the distance from the center of the FOV increases. Consequently, compact PET systems necessitate appropriate DOI information to achieve the desired spatial resolution. The measurement of DOI in pixelated-based PET detectors is typically conducted through two distinct methodologies: dual-ended and single-ended readout [90], [91]. In the dual-ended readout approach, photosensors are strategically positioned at both extremities of the scintillator array, enabling the determination of DOI by analyzing the signal amplitudes received by the dual photosensors. While the dual-ended readout technique affords continuous DOI measurements and superior DOI resolution, it requires the duplication of photosensors, readout electronics, and signal channels, posing practical challenges in compact geometries with high channel density, as anticipated in high-resolution dedicated PET scanners [92], [93], [94], [95].

Single-ended detectors offer a streamlined system architecture, potentially expanding their applicability to compact dedicated PET systems beyond the realm of medical applications. Phoswich detectors represent a well-established solution in this context. These detectors integrate multiple layers of scintillating crystals with distinct scintillation decay times, thereby enabling the discrimination of each layer through pulse shape analysis [96]. Alternative configurations include stratified structures wherein crystals are strategically positioned at varying spatial coordinates, allowing for identification through a detection map [97].

The light-sharing window (LSW) configuration constitutes an alternative single-ended readout methodology for DOI assessment, based on the alterations in the intercrystal reflector arrangements. This technique encodes DOI data into scintillation photon distributions, producing distinctive detection maps [98], [99]. In LSW-based single-ended readout DOI detectors, parameters such as the dimensions, geometry, location, and number of the windows significantly influence the photon distribution and the precision of DOI measurement. Recently, the Prism-PET design, incorporating a segmented prism light guide, has been employed to evaluate intercrystal light sharing and enhance DOI resolution [100], [101]. Nevertheless, the intricacy of the proposed systems renders their implementation in compact high-resolution systems exceedingly challenging. Digital signal processing emerges as a pivotal approach to addressing the DOI challenge without additional complexity. It has been demonstrated that an undersampled PET signal with up to 16 sampling points, predominantly concentrated on the rising edge, can discriminate the DOI due to the inherent timing characteristics of the pulses. This insight paves the way for digital signal processing techniques based on pulse digitization [102].

As previously discussed, monolithic crystals represent a viable solution to the DOI issue, as they inherently provide the three-dimensional interaction point position of incoming gamma rays. Despite the attractive high sensitivity and DOI capability, monolithic crystals are subject to edge effects that can significantly impact the precision of DOI measurements [103]. To address this issue, machine learning techniques and neural networks are employed to enhance location estimation performance in monolithic scintillator-based detector designs [104].

The final term in Eq. (1) represents the error which arises due to the imperfect collinearity of the two 511 keV gamma rays emitted following positron annihilation. While this term is not critical for compact scanners with a small radius designed for applications in zebrafish or crop science, it becomes significant in Positron Emission Particle Tracking (PEPT) large systems utilized for analyzing industrial flow processes. The impact of this error can only be mitigated through the design of appropriated system geometry, as it is an inherent characteristic of the PET imaging technique.

### 3.2 Need of dedicated processing and reconstruction

High-performance instrumentation alone is insufficient to guarantee the desired image quality, as PET is a tomographic technique that currently relies on statistical image reconstruction algorithms combined with mathematical models of the physical processes. Although many open-source software packages are available for PET reconstruction (e.g., [105], [106]), they may be inadequate for non-conventional scanner geometries, and their modeling capabilities are often limited. Regardless of the reconstruction algorithm, the accuracy of the models in representing signal formation and degradation phenomena is critical for achieving high image quality. To some extent, these models can compensate for image degradation induced by limited-view geometries, positron range, lack of DOI information, and inter-crystal scatter. In particular, accurate modeling is essential for cost-efficient small-diameter PET solutions without Time Of Flight (TOF) and DOI capabilities.

Corrections for attenuation, randoms, and scatter can be incorporated into the algorithms, as routinely done for clinical PET. However, some of these corrections require information about the object’s attenuation properties.

In the absence of additional information from CT, MRI or a transmission scan, other approaches have been proposed for clinical PET, being those based on the *Maximum likelihood reconstruction of attenuation and activity* (MLAA) approach [107], being the most popular ones. However, these techniques are not adequate for small-animal PET, as the lack of publications suggests. Possible reasons are low-count data and that the attenuation effects are small, leading to a weak signal to infer attenuation and thus making the algorithm unstable. A pragmatic approach used for rodent PET is to extract the outline of the attenuating object – the animal – from the emission image and, after segmentation, assign a uniform soft-tissue attenuation coefficient within this mask [108]. In the case of small aquatic animal PET with water-filled imaging chambers, it would be possible to generate a pre-computed attenuation map, assuming that the animal attenuation is equal to water. This approach might induce some bias in the swim bladder and proximal regions [21], and would require using fiducial marks or external information for alignment. Although their validation mainly focuses on clinical PET, recent developments in deep learning and AI-based attenuation correction might offer alternative solutions [109], [110], [111], for instance, by enabling the estimation of attenuation maps from limited anatomical information (as provided by optical cameras).

When sensitivity is an issue, e.g., in small-fish PET, a software solution to increase the number of useful counts consists in including inter-crystal scatter events into the reconstruction; these are events in which at least one of the two annihilation photons undergoes a Compton interaction, followed e.g. by photoabsoption [112]. While inter-crystal scatter has been long considered a minor issue, particularly in clinical PET, has seen renewed interest due to highly pixelated and multi-layer PET detectors, as these concepts are prone to Compton scattering. When the energy deposited in the first interaction is small and the energy window is narrow, these events remain unidentified while still contributing to detection uncertainty, as only the scattered photon is detected. On the other hand, the recovery of inter-crystal scatter events represents an additional source of events, even if it can come at the cost of some resolution degradation [112], [113]. To capture and exploit inter-crystal scatter events, large energy windows as well as dedicated coincidence sorting and processing are required. Two types of approaches have been suggested, either based on identifying the original photon trajectory before reconstruction (e.g. [112], [114], [115]), or through dedicated reconstruction including a model of the ambiguity in the detection [116]. A preliminary study has shown recently the potential of the latter approach for zebrafish PET imaging [117].

### 3.3 Time of Flight resolution becomes relevant only below 80 ps (FWHM)

The TOF measures the time difference between the detection times of the two annihilation gamma rays. The main contribution of TOF to image quality is in the Signal To Noise Ratio (SNR). The introduction of TOF in the tomographic image reconstruction algorithms improves the SNR of a factor described by the following formula [118]:

(2) $${\left(\frac{SNR_{TOF}}{SNR_{nonTOF}}\right)}^{2}=\frac{2D}{c\times CTR}$$

where c is the speed of light and D is the size of the target object. This relationship indicates that the Coincidence Time Rate (CTR) required to achieve a significant increase in the SNR is given by:

(3) $$CTR_{max}\left[ps\right]\approx \frac{200}{3}D\left[\mathrm{cm}\right]$$

Consequently, for imaging a small biological system with a characteristic dimension D<1 cm, as in case of zebrafishs, the minimum CTR required to achieve a substantial improvement in image SNR is approximately 70 ps. In the case of plants, whose characteristic tissue dimensions are on the millimetric scale, the required CTR is substantially decreased to only a few picoseconds.

The TOF resolution arises from the convolution of multiple contributions along the detection chain. Following the interaction of an incident gamma photon within a scintillation crystal, scintillation optical photons are generated. These photons propagate through the crystal towards the photosensor via a sequence of internal reflections and refractions, which modifies their effective path lengths and, consequently, their arrival times at the photodetector. As a result, the crystal geometry constitutes a critical determinant of the achievable TOF resolution. For instance, a degradation in TOF performance from 125 ps (FWHM) to 175 ps (FWHM) has been reported when a 3 × 3 mm^2^ Lutetium–Yttrium Oxyorthosilicate (LYSO) crystal with lengths varying between 5 mm and 20 mm is coupled to a SiPM readout [119]. The intrinsic temporal response and the surface finishing of the scintillator, the photon detection efficiency, as well as the timing characteristics and noise performance of the photodetector, together with the bandwidth limitations and timing jitter of the readout electronics, collectively contribute to the deterioration of the overall timing resolution of PET detectors [120]. Consequently, the best TOF resolution currently demonstrated at the system level lies in the range of 219 ps (FWHM) [121] to 249 ps (FWHM) [122]. Although current TOF performance remains advantageous for human imaging, where the characteristic dimensions of the biological target are on the order of several tens of centimeters, the calculations presented above indicate that the TOF capabilities of existing PET detectors are not sufficient for imaging small biological objects. For such applications, an improvement in timing resolution by at least one order of magnitude would be required to achieve a meaningful benefit. Still, even limited TOF capabilities might be of help to reduce the contribution of random and scatter events.

As reported in [118], [120], [123], ongoing research and development activities are focused on novel fast scintillator materials, such as metascintillators, photodetectors with on-chip integrated digital electronics, and fast-timing readout ASICs. These efforts aim to mitigate the present timing jitter and to approach the fundamental limits of the timing performance of PET detectors.

## 4 Systems and prototypes

### 4.1 PET systems for preclinical biomedical research beyond mice and rats

For well-established laboratory animals, such as mice and rats, there is a plethora of dedicated commercial and research PET scanner prototypes. Efforts in instrumentation have been invested in improving the resolution beyond 1 mm, increasing the FOV to allow for total-body PET, or allowing for multimodal imaging beyond CT and MRI. In parallel, research efforts are also directed towards developing more cost-efficient small-animal PET systems to support wider adoption in preclinical research. For further details, we refer the reader to recent reviews [124], [125], [126], [127], [128]. These advances also benefit the use of PET beyond mice and rats. The development of scanners with resolution below 0.5 mm might also open new possibilities for high-detail zebrafish and embryo imaging. For instance, the HR-PET [129], conceived for rodent neuroimaging, offers superb sensitivity at the center (1.02%) thanks to its small diameter (48 mm) and a ring covered by trapezoidal detector modules; each module consists of three layers of LYSO crystal arrays to allow for discrete DOI, with a crystal cross section of $0.7\times 0.7\;{\text{mm}}^{2}$ and lengths of 3 mm, 3 mm and 5 mm. Yet, the complexity of these scanners results in substantial cost.

PET technology is also well suited for in-vitro specimen imaging, which also requires a very high resolution and sensitivity [31]. However, the system efficiency can be lower than in in-vivo imaging, as the acquisition time is not a concern. Although some commercial products exist, many systems are usually research prototypes, and are often built with simplified geometry, reduced FOV, and inexpensive photodetectors to keep costs manageable. A common characteristic is the horizontal arrangement of the detectors to allow the sample to be placed flat at the center. Besides excised tissues, these systems are also of interest in investigating developing embryos.

In general, advances aimed at improving conventional small-animal PET, such as improved detector technology, very high sensitivity and submillimeter spatial resolution, can be transferred to highly specialized applications enabling higher image quality, better quantification, as well as more compact and cost-effective scanner designs. Some examples are human wrist PET, a 9-cm diameter commercial solution developed to determine the arterial input function for quantitative PET [130], and rodent paw PET imaging. The latter was proposed to study arthritis in preclinical models [131] and was composed of tiny Lutetium Oxyorthosilicate (LSO) crystals of size 0.43mm×0.43mm×8mm and an transaxial FOV of 16 mm.

A specific solution for PET imaging of zebrafish and small aquatic animals and, to our knowledge, the only one of its kind, is the *Multi-emission radioisotopes — marine animal imaging device* (MERMAID) [47], [56], [132]. This scanner prototype, currently under construction, consists of LYSO crystals of size 1.12×1.12×15.00 mm${}^{3}$, coupled one-to-one to SiPM units (Hamamatsu S13615-1050N-08); it features a very small diameter of approximately 66 mm to maximize sensitivity but also large enough to allow for the placement of a water-filled imaging chamber. However, decreasing the scanner diameter implies an increase in parallax errors and, subsequently, a worsening of the spatial resolution towards the edges of the FOV. Therefore, detection concepts with DOI are highly desirable for this application (see Section 3.1). In the same vein, some of the configurations proposed for PET-on-a-chip [64], with a detector-to-detector distance of 52 mm, can also be suitable for zebrafish imaging (and vice versa), not only because of the small thickness of the animals but also because of the activity levels (a maximum of a few megabequerel).

### 4.2 PET systems for plant imaging, crop imaging and digital precision agronomy

**Fig. 3:**
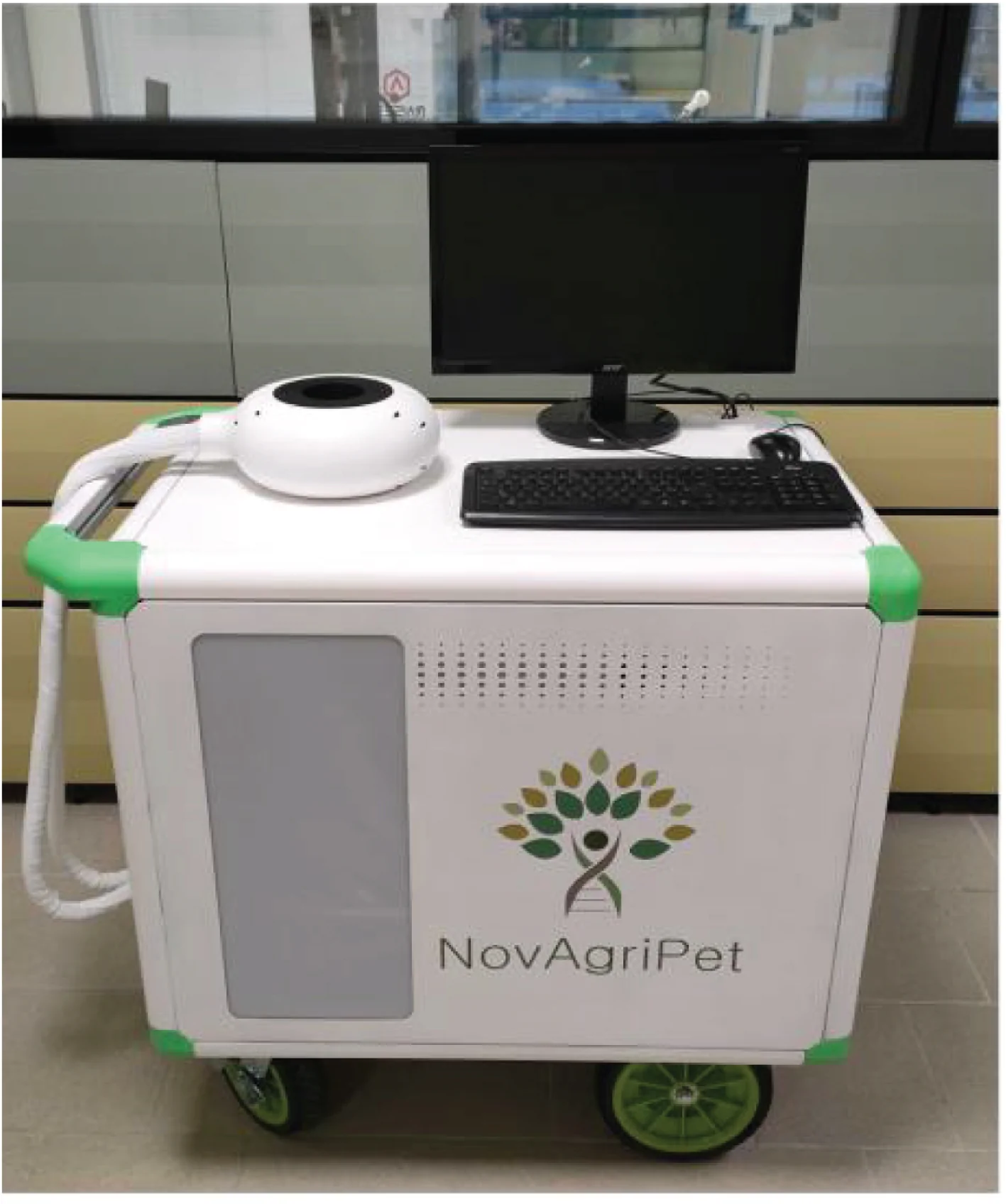
Example of AGRIPET, an All-Digital PET system developed for plant and crop imaging [133]. Figures of the other existing systems dedicated to plant imaging can be found in the following publications: PETIS [134], INVEON [135], phytoPET [136], OpenPET [137], PLANTIS [138], phenoPET [139].

Only a limited number of PET systems have been developed with architectures specifically optimized for plant imaging. These systems are summarized in Table 1. An example of a mobile plant PET system is shown in Fig. 3.

The AGRI-PET system [133] detection heads consist of a 6 × 6 array of LYSO scintillation crystals, each with a square cross section of 3.9 mm side length and an axial length of 20 mm. Each crystal is optically coupled in a 1:1 configuration to a SiPM (model FM30035, Onsemi), whose photosensitive area is also square, with a side length of 3.0 mm. The analog pulses generated by the SiPMs are digitized using a single Multi Voltage Threshold (MVT) channel. Each MVT board services two detector heads via a flat-cable connection. The prototype configuration comprises 20 MVT boards, which in total read out 40 detector heads. These heads are arranged into two semi-cylindrical modules, each composed of a 4 × 5 array of heads. The two modules can be mechanically separated to increase the transverse FOV. In this configuration, the system provides an axial FOV of 100.8 mm and a transverse FOV adjustable in the range of 80–120 mm. A central feature of the system is the MVT-based digital readout scheme. The analog waveforms are encoded using eight time samples corresponding to the threshold-crossing times at four distinct voltage levels. This approach enables a high-bandwidth, low-latency readout due to the reduced amount of transmitted data. The prototype has been experimentally evaluated to establish an initial proof of principle for in-field PET imaging, which, to the best of our knowledge, is demonstrated with AGRI PET for the first time. This achievement operationalizes the concept of crop imaging *from-lab-to-field*.

The PETIS scanner [134] is a compact planar positron imaging system installed within an environmental control chamber and is equipped with position-sensitive scintillation detectors based on a Bi4Ge3O12 (BGO) crystal detector module. The system consists of two opposing detector heads, each comprising a 4 (columns) × 6 (rows) array of block-detector units. Each block-detector unit contains a 10 × 10 array of BGO scintillator elements that are optically coupled to a metal-packaged position-sensitive photomultiplier tube (PS-PMT; Hamamatsu R8520–00-C12, Hamamatsu Photonics Co., Shizuoka, Japan). Each BGO crystal element has a cross-sectional area of 2 mm × 2 mm and a length of 20 mm. The overall dimensions of each detector head are 285 mm (height) × 200 mm (width) × 200 mm (length). The two detector heads are mounted face-to-face with an inter-detector distance of 200 mm. The detector pair records coincident 511 keV annihilation photons using a standard coincidence detection method to reconstruct planar focal images. Coincidence events are registered between a given block-detector unit and a 3 × 3 array of block-detector units positioned directly opposite it. Each image has a FOV of 141.9 mm (width) × 214.5 mm (height), with a matrix size of 129 × 195 pixels. The in-plane spatial resolution at the center of the FOV is 2.3 mm full width at half maximum (FWHM). The PETIS scanner is historically recognized for enabling the first quantitative modeling studies in plant science based on time-resolved kinetic PET methodologies [140]. PETIS-derived datasets have been employed to determine model-independent transfer functions for ^11^C translocation in crop species. In sorghum, statistically significant correlations were identified between the mean velocity of ^11^C transport and NaCl exposure, with observed transport rates ranging from 0.7 to 1.8 cm min^−1^ [141]. Likewise, after administering ^11^C—CO_2_ to an intact broad bean (Vicia faba L.) plant, it was possible to estimate both the average flow velocities and the partitioning ratios of photoassimilates among the individual nodes and internodes along the analyzed stem [142]. Broadening the application of PETIS to additional physiological processes, real-time translocation dynamics of iron (Fe) in barley (Hordeum vulgare L. cv. Ehimehadaka no. 1) were visualized using the positron-emitting tracer ^52^Fe [143], and cadmium (Cd) accumulation patterns were characterized in the Cd hyperaccumulator fern Athyrium yokoscense (Ay) and in tobacco (Nicotiana tabacum, Nt) [144]. The immediate agronomic relevance of these imaging approaches was demonstrated in studies aimed at enhancing the efficiency of carbon translocation during tomato fruit production [145]. The primary limitations of the PETIS system are its restriction to two-dimensional imaging and its limited portability for in-field deployment. Consequently, PETIS has primarily served as a platform for demonstrating laboratory-based PET imaging capabilities in crop science.

Adaptability to diverse plant morphologies constitutes a key design criterion for PET systems intended for crop imaging. As an illustrative example, a single scanner has been configured to incorporate two distinct types of detector modules with different geometries, thereby addressing a range of imaging requirements [135]. The first detector type comprises microPET modules, each incorporating four PS-PMTs that read out four LSO scintillator arrays. Each LSO array consists of an 8 × 8 matrix of crystals, each crystal measuring 2.2 mm × 2.2 mm × 10 mm with a 2.4 mm pitch. For imaging larger plants, 21 such microPET modules are assembled to form a detector panel that provides a large solid-angle coverage. The second detector type consists of eight Siemens Inveon modules, each of which also contains four PS-PMTs coupled to four LSO crystal arrays. In this configuration, each LSO array comprises a 20 × 20 crystal matrix, with individual crystals measuring 1.51 mm × 1.51 mm × 10 mm and arranged with a 1.59 mm pitch. The system can be reconfigured into multiple geometric arrangements to accommodate plants of varying sizes and architectural forms.

**Table 1:** Overview of PET systems and prototypes designed and in use for plant imaging.

Observable	AGRI PET	PETIS		PHYTOPET	OpenPET	PLANTIS	phenoPET
	[133]	[134]	[135]	[136]	[137]	[138]	[139]
Shape	two	planar	variable	modular	two	planar	cylinder
	half-cylinders				cylinders		

Orientation	vertical	vertical	vertical	vertical	horizontal	vertical	vertical

Crystal	3.9	2.0	1.5	1.0	2.9	2.0	1.85
size [mm]			2.2				

Crystal	20	20	10	10	5	10	10
length [mm]							

Axial	100.8	285	80–120	variable	110	101	195
FOV [mm]							

Transverse	83.4-120	200	150–400	variable	84–126	73	255
FOV [mm]							

Sensitivity	16.9–9.1	n.a.	4.3–1.3	n.a.	8.7–6.7	3.7	3.6
[%]							

Spatial	1.93–3.11	2.3	1.25	3	1.5–2.2	1.39	0.89
resolution [mm]							

Imaging	3D	2D	3D	3D	3D	3D	3D

DOI	no	no	no	no	yes	yes	no

PhytoPET [136] is constructed from detector modules comprising individual 5 cm × 5 cm Hamamatsu H8500 PS-PMTs. Each H8500 PSPMT is optically coupled to a 48 × 48 element LYSO:Ce scintillator array, 10 mm in thickness, with a 1.0 mm pitch, custom-fabricated by Proteus Inc. (Chagrin Falls, OH). Each PhytoPET detector module has external dimensions of 120 mm × 60 mm × 60 mm (L × W × H). The modules can be vertically stacked to accommodate a range of plant morphologies. Each module is mechanically designed such that, upon stacking, it is kinematically constrained and locked into position via a complementary set of three detents and bonded metal spheres. Because optical occlusion can significantly perturb plant physiological processes, the detector housing employs a specularly reflective finish, thereby minimizing unintended light blockage. PhytoPET has been used for lab-based imaging of corn sugar translocation [146], [147].

Full coverage of the entire plant is not required. Recently, the OpenPET prototype has been employed to image and quantify sugar translocation into the fruit of a strawberry plant [137], [148]. The system consists of two detector rings with an inner diameter of 110 mm, each composed of eight block detectors, mounted with a variable axial gap. Each block detector, which provides four-layer DOI capability, consisted of a 14 × 14 × 4-layer array of Lu_1.8_Gd_0.2_SiO_5_:Ce (LGSO) scintillator crystals (Hitachi Chemical Co., Japan), with individual crystal dimensions of 2.9 × 2.9 × 5.0 mm${}^{3}$. The array was optically coupled to a 64-channel H8500 photomultiplier tube (PMT; Hamamatsu Photonics KK, Japan). The axial length W of the crystal blocks was 42 mm. The axial gap G between opposing crystal blocks was also set to 42 mm, which is the maximum value that yields an axially continuous FOV.

DOI information is therefore a critical parameter in crop imaging applications. The PLANTIS system [138] employs multichannel photomultiplier tubes (H7600-00-M64, Hamamatsu), each providing readout for 64 scintillator pixels. Each individual pixel consists of a phoswich configuration comprising LSO and lutetium–yttrium aluminosilicate (LuYAP) crystals, enabling the determination of DOI. Every scintillator pixel is directly optically coupled to a single photomultiplier tube channel. The crystals are optically polished and embedded in BaSO_4_ matrices to ensure mechanical stability and to enhance scintillation light collection.

Finally, the phenoPET system features a vertically oriented FOV with a radius of 90 mm and a height of 202 mm [139]. The FOV is covered by 36 detector modules, which are arranged into twelve sectors, yielding a characteristic configuration of crystal matrices. The distance between opposing sectors is 255 mm. A central innovation of this system is that each detector module comprises a 2 × 2 array of digital photon counters (DPCs) (type: DPC3200-22-44, Philips Digital Photon Counting 2015), hereafter referred to as a tile. Each tile consists of an 8 × 8 array of digital SiPM coupled to a 16 × 16 matrix of LYSO crystals (Crystal Photonics, Inc., Sanford, Florida 32773). The individual LYSO crystals have dimensions of 1.85 mm × 1.85 mm × 10 mm. A combination of transparent and opaque optical coupling enables separation of the four crystals positioned above each SiPM. The system is used for ^11^C—CO_2_ radiolabeling of crops in controlled “in-lab” experiments.

## 5 Outlook and conclusions

Functional imaging—and PET in particular—is emerging as a key methodology for applications that extend well beyond the clinical indications for which it was initially developed. Environmental monitoring, climate change research, agriculture, and food security represent novel and rapidly expanding domains of impact for these technologies. Prospectively, the combination of targeted radiotracer approaches with high-resolution PET in aquatic organisms offers substantial potential for tracking micro- and nanoplastics in vivo in relevant animal models. This will enable detailed characterization of their biodistribution, kinetics, and biological effects, as already demonstrated in murine models [149], [150]. In a similar manner, the implementation of PET in agricultural systems is expected to facilitate more sustainable food production practices, for example by optimizing nitrogen fertilizer usage and improving water management, thereby reducing the environmental footprint of crop cultivation.

Emerging digital sensor technologies are expected to drive the development of the next generation of compact, high-resolution, high-sensitivity, mobile, and low-power PET systems. In particular, the digital silicon photomultiplier is anticipated to play a prominent role, owing to the monolithic integration of photosensors and digital electronics on a single substrate, which enables intelligent on-chip signal processing and readout [34].

The integration and synergistic use of diverse imaging modalities represents a further key driver of innovation. Specifically, the multimodal combination of PET, MRI, and optical microscopy, implemented in mobile, compact platforms suitable for deployment in a wide range of environments, including field settings, will enable complementary interrogation of functional and morphological signatures. The recent Horizon Europe European Innovation Council Pathfinder project *RE IMAGINE CROPS* [151] exemplifies this trend by introducing a novel technology that combines PET with endoscopic optical microscopy. This is achieved through a multimodal tracer based on a fluorescent protein labeled with the positron-emitting radionuclide ^89^Zr, thereby enabling in-field multimodal imaging of crops.

The renewed scientific interest in imaging applications beyond the clinical and medical domains is opening new market opportunities for PET and functional imaging more broadly, defining emerging research directions and driving technological development towards ever higher spatial resolution and sensitivity. Emerging new research applications of PET are consequently becoming key driving factors in the development of next-generation PET technologies.

## CRediT authorship contribution statement

**Nicola D’Ascenzo:** Writing – original draft. **Magdalena Rafecas:** Writing – original draft.

## Declaration of competing interest

The authors declare that they have no known competing financial interests or personal relationships that could have appeared to influence the work reported in this paper.
